# Home treatment for adolescents with eating disorders as an add-on to family based therapy

**DOI:** 10.1192/j.eurpsy.2021.331

**Published:** 2021-08-13

**Authors:** N. Flütsch, D. Pauli

**Affiliations:** Department Of Child And Adolescent Psychiatry And Psychotherapy, University of Zurich, Zurich, Switzerland

**Keywords:** eating disorders, home treatment, adolescents, family based treatment

## Abstract

**Introduction:**

Family-based therapy (FBT) has been proven effective in treating eating disorders among children and adolescents. However, many families have difficulties implementing the measures recommended in outpatient therapy.

**Objectives:**

This study examines the effectiveness of add-on home treatment (HT) to family based therapy (FBT) in adolescents with anorexia nervosa (AN). The HT intervention is delivered by specialized nurses and aims at supporting patients and parents to re-establish family meals in the home environment.

**Methods:**

We performed an case-control study in AN patients comparing 44 (42 female, 2 male) adolescents receiving FBT augmented with HT compared to 22 (22 female, 1 male) participants receiving FBT alone. Eating disorder diagnosis, psychopathology and severity of clinical symptoms were assessed using (EDE, EDI-2) and clinical parameters (BMI, menstrual status, level of over-exercising) at baseline and after 3-months.

**Results:**

After 3 months both treatment groups showed a significant early weight gain, a reduction in the rate of AN diagnoses assessed with the EDE interview and a reduction in EDI-2 total scores. The combined HT/FBT group showed a significantly greater increase in BMI than the FBT-only group. In the combined HT/FBT group none of the patients had to be admitted to hospital while 13.6% of the FBT-only group had to be referred to inpatient treatment. Treatment satisfaction in the combined HT/FBT group was high in both patients and parents.

**Conclusions:**

Our results suggest that HT augmented FBT is superior compared to FBT alone in terms of early weight gain and might reduce the risk of hospital admission in adolescent AN.

**Disclosure:**

No significant relationships.

